# Overexpression of MnSOD Protects against Cold Storage-Induced Mitochondrial Injury but Not against OMA1-Dependent OPA1 Proteolytic Processing in Rat Renal Proximal Tubular Cells

**DOI:** 10.3390/antiox10081272

**Published:** 2021-08-11

**Authors:** Julia Tobacyk, Grishma KC, Lee Ann MacMillan-Crow

**Affiliations:** Department of Pharmacology and Toxicology, College of Medicine, University of Arkansas for Medical Sciences, 4301 W. Markham Street, Mail Slot 611, Little Rock, AR 72205, USA; JTOBACYK@uams.edu (J.T.); GKc@uams.edu (G.K.)

**Keywords:** mitochondria, kidney transplantation, cold storage, oxidative stress, OPA1, OMA1, mitochondrial fusion

## Abstract

Kidneys from deceased donors undergo cold storage (CS) preservation before transplantation. Although CS is a clinical necessity for extending organ quality preservation, CS causes mitochondrial and renal injury. Specifically, many studies, including our own, have shown that the triggering event of CS-induced renal injury is mitochondrial reactive oxygen species (mROS). Here, we explored the role of OMA1-depedent OPA1 proteolytic processing in rat kidney proximal tubular epithelial (NRK) cells in an in vitro model of renal CS (18 h), followed by rewarming (6 h) (CS + RW). The involvement of mROS was evaluated by stably overexpressing manganese superoxide dismutase (MnSOD), an essential mitochondrial antioxidant enzyme, in NRK cells. Western blots detected rapid OPA1 proteolytic processing and a decrease in ATP-dependent cell viability in NRK cells subjected to CS + RW compared to control cells. Small interfering RNA (siRNA) knockdown of OMA1 reduced proteolytic processing of OPA1, suggesting that OMA1 is responsible for OPA1 proteolytic processing during CS + RW-induced renal injury. Overexpression of MnSOD during CS + RW reduced cell death, mitochondrial respiratory dysfunction, and ATP-dependent cell viability, but it did not prevent OMA1-dependent OPA1 processing. These data show for the first time that OMA1 is responsible for proteolytically cleaving OPA1 in a redox-independent manner during renal cell CS.

## 1. Introduction

More than 500,000 people in the United States live with end-stage kidney disease (ESKD) [1] and kidney transplantation is the optimal treatment for ESKD. Cold storage (CS) preservation is a critical step during the process of transplanting kidneys from deceased donors and it is designed to lower the metabolic rate, maintain organ viability, and provide valuable time to find a matched recipient [2,3,4,5]. Even though CS has increased the number of available kidney donors, CS followed by reperfusion during transplantation is associated with renal injury [6,7,8]. Thus, patients who receive kidneys from deceased donors have impaired long-term transplant success and more complications than those transplanted from live donors, which avoid the CS process [9,10]. Despite these worse outcomes, there are few, if any, clinical interventions to protect transplanted kidneys from CS-induced injury; making it imperative to identify new therapies to alleviate CS-induced injury and improve overall graft outcome.

Numerous studies using in vivo and in vitro translational models, including our own, have shown that mitochondrial injury and elevation of mitochondrial reactive oxygen species (mROS) are key events during CS-induced renal injury [11,12,13,14,15,16,17,18]. This current study is based on our previous report showing severe mitochondrial injury in our renal rat transplant model [19]. Furthermore, CS followed by transplantation induced OPA1 proteolytic processing and aberrant OMA1 expression in vivo, which lead us to hypothesize that OMA1-dependent OPA1 proteolytic processing plays an integral role in CS-induced renal injury. Thus, the goal of the current study is to further investigate the mechanistic role of OPA1 in our rat renal cell model of CS preservation.

In our previous report, we show that impaired mitochondrial fission and fusion may be a contributing factor to CS-induced renal injury [19]. Furthermore, abnormalities in mitochondrial fission and fusion have been implicated in a number of human diseases, including neurodegenerative and cardiovascular diseases [20,21,22,23]. Fission and fusion are two opposing forces that are crucial to mitochondrial regulation and are connected to cell cycle regulation, quality control, and bioenergetics [20,24]. Mitochondrial fusion is predominantly regulated by a GTPase called Optic Atrophy Protein type 1 (OPA1), which participates in fusion of the inner mitochondrial membrane. OPA1 exists in its long (L-OPA1) and short (S-OPA1) form, where the balance of both the L- and S-OPA1 promote efficient fusion, whereas accumulation of the S-OPA1 may promote fission, leading to mitochondrial fragmentation [25,26]. There are two zinc metalloproteases located in the inner mitochondrial membrane that participate in OPA1 proteolytic processing—YME1L and OMA1 [27,28]. The goal of the current study is to further dissect the role that mROS has on OPA1, using our well-established rat renal proximal tubular (NRK) cell model where NRK cells are exposed to CS followed by rewarming (CS + RW) [15,17,29,30]. These prior studies utilized mitochondrial targeted antioxidants as well as overexpression of manganese superoxide dismutase (MnSOD), the primary mitochondrial antioxidant enzyme, in NRK cells, as additional evidence supporting generation of mROS during CS.

Here, we show that OMA1 plays a role in OPA1 proteolytic processing during CS + RW in NRK cells. Overexpression of MnSOD reversed the pathophysiology associated with CS + RW-induced cellular and mitochondrial injury, but it did not prevent OPA1 proteolytic cleavage, suggesting that these are two independent pathways involved in CS + RW-induced cytotoxicity. A deeper understanding of molecular pathways related to renal CS-induced injury could benefit designing specific pharmacological agents and therapies to improve kidney transplantation from deceased donors.

## 2. Materials and Methods

### 2.1. Cell Culture and Cold Storage/Rewarming Model

Normal rat kidney proximal tubular cell line (NRK-52E; American Type Culture Collection; no. CRL-1571) were maintained in Dulbecco’s Modified Eagle Medium (DMEM; Gibco) containing 5% heat-inactivated fetal bovine serum (FBS; Gibco) and 1% penicillin/streptomycin (Gibco) in a 5% CO2 incubator at 37 °C. MnSOD overexpressing NRK cells were generated by stably transfecting with the pMnSOD plasmid DNA (10 μg) using lipofectamine as previously described [31]. The pMnSOD-containing cells were selected using G418. MnSOD overexpression was confirmed using MnSOD activity assay utilizing the cytochrome c reduction method as described by McCord and Fridovitch [32]. OMA1 wild type (WT) and knockout (KO) primary mouse embryonic fibroblasts (MEF) were kindly provided by Dr. Pedro Quirós (Hospital Universitario de Puerto Real, Spain). MEF were grown in complete cell culture medium containing DMEM, 10% heat-inactivated FBS, and 1% penicillin/streptomycin. OMA1 knockdown was confirmed via western immunoblotting.

**Figure 1:**
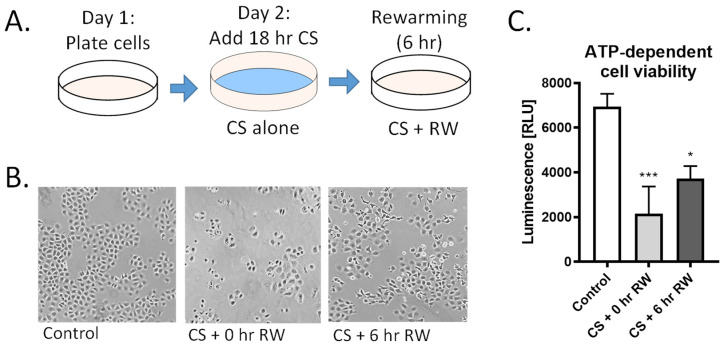
CS + RW induces NRK cell injury. (**A**) NRK cells were exposed to CS for 18 h in UW solution followed by 6 h of RW. (**B**) Representative images of microscopic evaluation used for qualitative assessment of cell injury after CS and CS + RW. After 18 h, CS followed by 0 h and 6 h RW, NRK cells exhibited altered cell morphology indicated by stretched, rounded, and swollen cells. (**C**) Cytotoxicity was assessed by a luminescent cell viability assay. ATP-dependent viability was decreased in both CS and CS + RW compared to control. One-way ANOVA followed by Tukey’s post-hoc test. *n* = 5; ±SEM. * *p* < 0.05, *** *p* < 0.001.

For CS alone, NRK cells were placed in CS solution (University of Wisconsin/Viaspan) at 4 °C for 18 h. For cold storage followed by rewarming (CS + RW) treatment, cells were placed in CS solution for 18 h at 4 °C followed by removal of the UW solution, washing cells three times with cold PBS, and adding cold, complete cell growth medium and, lastly, incubating cells at 37 °C for 6 h (Figure 1A). Moreover, 18 h CS and 6 h RW were selected as clinically relevant endpoints that mimic CS and reperfusion injury in a rat kidney transplant model [33]. Untreated NRK cells were used as controls.

### 2.2. SiRNA Transfection

At 50% confluency, NRK cells were transiently transfected with 50 nM OMA1 (siGENOME SMARTpool, Dharmacon; target sequence: GUAGGACUCUCAAGAACAA; UUGAAUAGCCUUCGUGCUU; GACAUACGCACUUGGAAA; GGCAAUGCCUUCGUGCUU) using siRNA transfection reagent (Invitrogen, Waltham, MA, USA) in OPTI-MEM (Invitrogen) with Lipofectamine RNAiMAX and Lipofectamine 2000 for 24 and/or 48 h at 37 °C as previously described [34]. The same concentration of scrambled siRNA (ON-TARGET plus Control Pool, non-targeting pool, Dharmacon; target sequences: UGGUUUACAUGUCGACUAA, UGGUUUACAUGUUGUGUGA, UGGUUUACAUGUUUUCUGA, UGGUUUACAUGUUUUCCUA) was used as a control. The next day, CS + RW or control treatments were assigned. Efficiency of OMA1 transfection and knockdown was assessed by western blotting as previously described [34].

### 2.3. Protein Lysates and Western Blotting

Protein lysates from NRK cells were prepared using radioimmunoprecipitation assay (RIPA) buffer (Sigma, Ronkonkoma, NY, USA) composed of 1 mM phenylmethylsulfonyl fluoride, 1 mM 1,4-dithiothreitol, and 1× Halt™ protease and phosphatase inhibitor cocktail (Thermo Scientific, Waltham, MA, USA). Protein concentrations were determined by Coomassie Plus™ protein assay reagent (Thermo Scientific). Protein lysates (25 μg) were resolved via SDS-PAGE (200 V; 27 min) using precast Bolt™ 8% Bis-Tris Plus gels (Invitrogen, Waltham, MA, USA) followed by wet-tank electrophoretic transfer (100 V; 2 h) to a 0.2 μm polyvinylidene difluoride (PVDF) membrane (Bio-Rad, Hercules, CA, USA). Following transfer, membranes were blocked with 5% non-fat dry milk in TBS-T (0.05% Tween-20) for 45 min at room temperature, except for western blots probed with OMA1; the membranes were blocked with 5% BSA in TBS-T. The membranes were incubated with the following antibodies: actin (1:1000, Sigma, #A5441), YME1L (1:1000, Abgent, #AP4882a), OMA1 (1:1000, Santa Cruz, # sc-515788) in 5% BSA, OPA1 (1:1000, Abcam, #ab42364) overnight at 4 °C. Actin was used as a loading control and SeeBlue2 Pre-stained Protein standard was used as a marker (Thermo Scientific). The next day, probed membranes were washed three times with TBS-T for 5 min and incubated for 45 min at room temperature with horseradish peroxidase-conjugated secondary antibodies (1:30,000), washed three times with TBS-T for 5 min each, and imaged using chemiluminescence (SuperSignal West Pico PLUS Chemiluminescent Substrate, Thermo Scientific). Densitometric analyses of Western blots were performed using AlphaEaseFC software (Alpha Innotech, San Leandro, CA, USA), which provided a semi-qualitative assessment.

### 2.4. Measurement of NRK Cell Cytotoxicity

The CellTiter-Glo^®^ Luminescent Cell Viability Assay Kit (Promega, Madison, WI, USA) was used to determine the number of viable cells. This assay utilizes the luciferase reaction to measure ATP, a global indicator of metabolically active cells. Cells (5000 for control wells and 10,000 for CS + RW wells) were plated into clear 96-well plates. All treatments were performed in triplicates. ATP-dependent cell viability was measured as per manufacturer’s instructions.

### 2.5. High-Resolution Respirometry

The activity of mitochondrial respiratory complexes in digitonin-permeabilized NRK cells was measured using high-resolution respirometry (Oxygraph-2k, Oroboros Instruments, Innsbruck, Austria) according to the substrate-inhibitor-titration protocol previously described [16,35,36] with minor modifications. Briefly, NRK cells (4–7 × 10^6^) were incubated for 15 min at 4 °C with digitonin (50 µg/mL), prepared in MiRO5 buffer (60 mM K-lactobionate; 0.5 mM EDTA; 3 mM MgCl2; 20 mM taurine; 10 mM KH2PO4; 20 mM HEPES; 110 mM BSA; 1 g/L sucrose, pH 7.0) under nutating conditions, followed by one rinse with cold MiRO5 buffer. Mitochondrial respiration was initiated by adding complex I substrates: 2 mM malate and 10 mM glutamate, followed by 5 mM ADP to achieve State 3 respiration. Next, 0.5 µM Rotenone was added to inhibit complex I. To measure complex II and complex III respiration, 10 mM succinate was added, followed by 2 mM malonic acid to inhibit complex II respiration and 5 µM antimycin A to inhibit complex III. Complex IV respiration was initiated by adding 50 µM tetramethyl-p-phenylenediamine (TMPD; ascorbate stabilized) and inhibited using 250 mM Azide. Data analysis was performed with DatLab 4.2 software (Oroboros, Innsbruck, Austria) and cellular respiration of each individual mitochondrial complex was expressed as oxygen flux (pmol/s*Million Cells).

### 2.6. Statistical Analysis

Results are presented as the mean ± standard error of the mean (±SEM). All analyses were performed using GraphPad Prism, version 8 (GraphPad Prism software, USA). Data from multiple group comparisons (control vs. 1–6 h RW) were analyzed with a one-way ANOVA followed by Tukey’s post-hoc test. An unpaired Student’s t-test was used when comparing differences between the means of two groups (control NRK vs. MnSOD OE +/− CS + RW; MEF OMA1 WT vs. MEF OMA1 KO +/− CS + RW). Data from NRK cells transfected with siRNA and treated with CS + RW were analyzed with a two-way ANOVA followed by the Sidak multiple comparison test. The main effects include Treatment (control vs. CS + RW), and Type of SiRNA (scramble vs. OMA1 siRNA) as well as the interaction effect. All differences with *p* < 0.05 were considered statistically significant.

## 3. Results

### 3.1. CS and CS + RW Induces NRK Cell Injury

We have previously reported that renal CS, followed by transplantation using a rat model, induces mitochondrial injury and impairment of mitochondrial fusion and fission proteins [19]. Here, we further investigated the impact of CS + RW on renal proximal tubule cell function, cell morphology, and ATP-dependent cell viability. NRK cells were exposed to 18 h CS alone or to CS + RW (18 + 6 h, respectively; Figure 1A). NRK cells exposed to 18 h CS ‘only’ exhibited rounded and constricted morphology with free-floating dead cells (Figure 1B). Furthermore, CS alone induced a 69% decrease in ATP-dependent cell viability compared to control/untreated cells (Figure 1C). Similarly, CS + RW treatment lead to an altered cell morphology and a 46% decrease in ATP-dependent cell viability compared to control/untreated cells (Figure 1C). These reports are consistent with our published study that 24 h CS followed by 6 h RW leads to 40% NRK cell death [15].

### 3.2. Rapid Proteolytic Processing of OPA1 Occurs during CS + RW in NRK Cells

**Figure 2:**
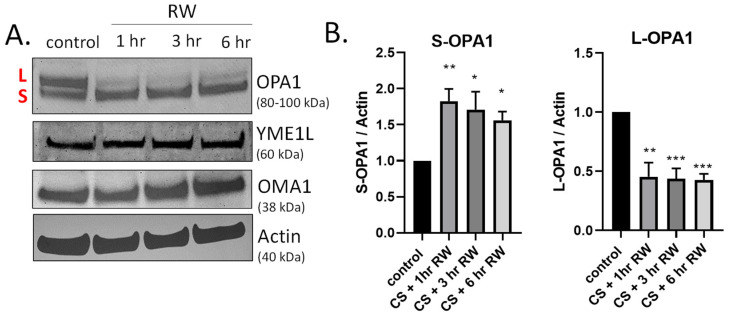
Time course of OPA1 proteolytic processing during CS + RW. (**A**) Representative western blots (where each lane is loaded with 25 μg NRK cell lysate protein) show OPA1 proteolytic processing, evidenced by loss of long form or L-OPA1 (~100 kDa) and increased short form or S-OPA1 (~80 kDa) as early as 18 h CS + 1 h RW in NRK cells. CS + RW did not alter protein expression of YME1L (~60 kDa) or OMA1 (~38 kDa). (**B**) Densitometric analysis using actin as a loading control shows that 18 h CS + 1, 3, and 6 h RW significantly increased S-OPA1 and decreased L-OPA1. Data were evaluated using one-way ANOVA followed by Tukey’s post-hoc test. * *p* < 0.05, ** *p* < 0.01, *** *p* < 0.001. *n* = 4 independent experiments were performed. Data are shown as mean density ±SEM (bars; arbitrary units).

OPA1 is a GTPase that controls key mitochondrial functions, including mitochondrial fusion, bioenergetics, mtDNA maintenance, and cristae integrity [22,37,38]. During both normal homeostasis and cellular stress responses OPA1 undergoes proteolytic processing by two inner-membrane proteases, YME1L and OMA1, leading to accumulation of both long and short forms of OPA1 [27,39,40,41,42]. We have previously published that OPA1 is proteolytically processed (loss of L-OPA1 and increased S-OPA1) in our in vivo rat kidney transplantation model [19]. This earlier study utilized a 24 h rewarming/post-transplantation time point. Here, we aimed to determine the kinetics of OPA1 proteolytic processing after shorter RW time points (1–6 h) utilizing the in vitro NRK cell model. Interestingly, OPA1 western blots show extensive processing as early as 1 h RW (Figure 2A). Densitometric analysis revealed a significant decrease in L-OPA1 and increase in S-OPA1 after all time points of RW, compared to control/untreated NRK cells (Figure 2B). These data suggest that OPA1 proteolytic processing occurs early during RW. Additionally, OPA1 was cleaved from its L-OPA1 to S-OPA1 form during 18 h CS alone (data not shown). Interestingly, the expression of YME1L and OMA1 did not change with CS + RW (Figure 2A).

### 3.3. CS + RW Induces OMA1-Dependent OPA1 Proteolytic Processing in NRK Cells

**Figure 3:**
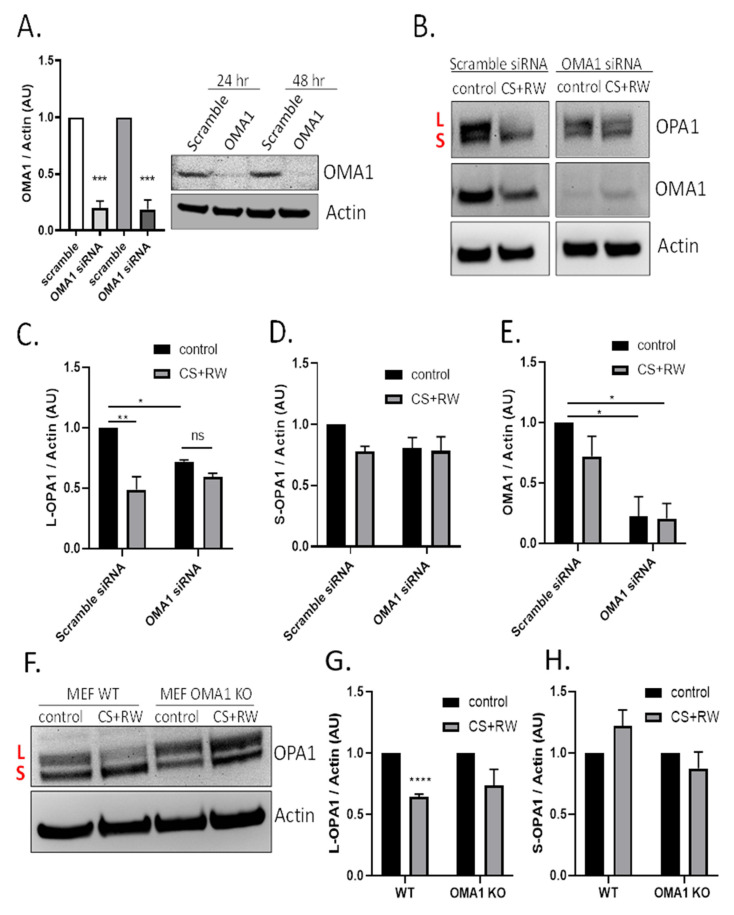
CS + RW induces OMA1-dependent OPA1 proteolytic processing in NRK cells. (**A**) Representative OMA1 western blots of NRK cells transfected with 50 nM of OMA1 or Scramble Scheme 80. and 79% at 24 and 48 h, respectively. (**B**) Representative OPA1, OMA1, and actin western blots after transfection (Scramble vs. OMA1 siRNA) combined with CS + RW showing enhanced OPA1 proteolytic processing with Scramble transfection, which was prevented in OMA1 siRNA transfected NRK cells. (**C**–**E**) Densitometric analysis using actin as a loading control shows significant loss of L-OPA1 (~100 kDa) in scramble siRNA combined with CS + RW in NRK cells (**). NRK cells transfected with OMA1 siRNA combined with CS + RW showed no significant difference in L or S-OPA1. However, L-OPA1 was significantly reduced in control OMA1 siRNA transfected NRK cells (*). OMA1 expression was significantly reduced in OMA1 siRNA transfected NRK cells with our without CS + RW. (**F**) Representative OPA1 and actin western blot in MEF cells after CS + RW. (**G**,**H**) Densitometric analysis using actin as a loading control shows significant loss of L-OPA1 in WT cells exposed to CS + RW (***), which was blocked in OMA1 KO cells. No significant changes in S-OPA1 expression were observed. Data were evaluated using unpaired t-test and two-way ANOVA followed by the Sidak post-hoc test as described in Methods. * *p* < 0.05, ** *p* < 0.01, *** *p* < 0.001, and **** *p* < 0.0001. *n* = 3–4 independent experiments were performed. Data are shown as mean density ±SEM (bars; arbitrary units).

The next experiments determined whether the mitochondrial protease OMA1 was involved in OPA1 proteolytic processing during CS + RW. NRK cells were first transfected with 50 nM of OMA1 siRNA and knockdown efficiency was confirmed via immunoblotting. OMA1 siRNA transfection resulted in 80% knockdown at 24 h and 79% knockdown after 48 h compared to cells transfected with scramble siRNA (Figure 3A). Next, NRK cells were transfected with OMA1 siRNA or scramble siRNA and, then CS + RW was initiated 24 h post-transfection. A representative OPA1 western blot shows that OMA1 knockdown appeared to prevent OPA1 processing (Figure 3B). Densitometric analysis using a two-way ANOVA (F(1, 8) = 31.90, *p* = 0.0005) revealed a significant decrease in L-OPA1 expression after CS + RW in NRK cells transfected with scramble siRNA, which was blocked in NRK cells transfected with OMA1 siRNA. However, unexpectedly, L-OPA1 was also reduced after OMA1 siRNA transfection alone (F(1, 8) = 11.43, *p* = 0.0096)) (Figure 3C). In addition, no significant changes were observed with S-OPA1 in NRK cells that had undergone transfection followed by exposure to CS + RW (Figure 3D). To verify the requirement for OMA1 during CS + RW-induced OPA1 processing, we also used the mouse embryonic fibroblast (MEF) wild type and OMA1 knockout cell model. Similar to results in the NRK cell model, western blotting showed that L-OPA1 was reduced after CS + RW, which was blocked in MEF OMA1 KO cells (Figure 3F–H). In summary, CS + RW leads to increased OMA1-mediated L-OPA1 processing, but the role of S-OPA1 proteolytic processing needs further experimentation to fully understand.

### 3.4. MnSOD Overexpression Does Not Prevent OMA1-Dependent OPA1 Proteolytic Processing during CS + RW

**Figure 4:**
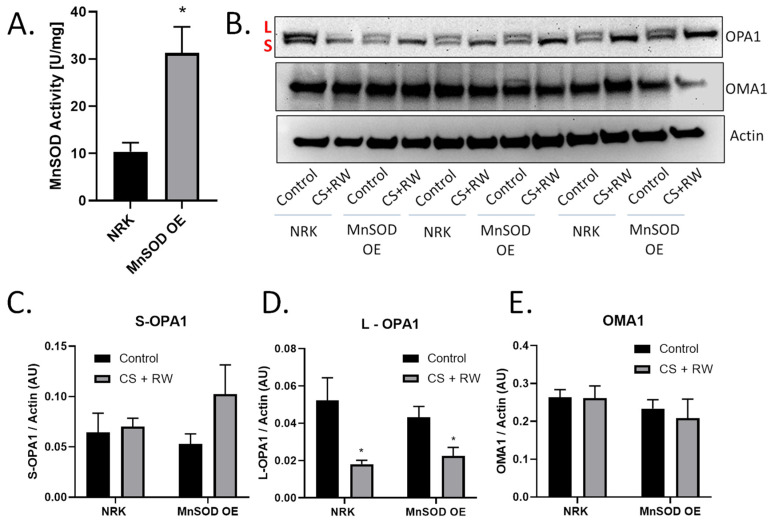
Overexpressing MnSOD did not prevent CS + RW-induced OPA1 proteolytic processing. (**A**) MnSOD activity assay utilizing cytochrome C reduction showed a threefold increase in MnSOD activity in NRK cells stably overexpressing MnSOD (MnSOD OE) compared to control/untransfected NRK cells. (**B**) Representative western blot of OPA1, OMA1, and actin (loading control) from control or CS + RW treated NRK cells and NRK cells overexpressing MnSOD. (**C**–**E**) Densitometry values were normalized to actin (~40 kDa) and expressed as mean density ±SEM (bars; arbitrary units). Data were evaluated using unpaired student t-test, * *p* < 0.05, *n* = 3 separate experiments. Abbreviations: AU—arbitrary units, L—long-OPA1, S—short-OPA1, MnSOD OE—manganese superoxide dismutase overexpression.

Previous reports revealed that generation of mitochondrial ROS (mROS) contributes to CS + RW-induced cell injury [15,29,30,33]. MnSOD is the primary mitochondrial antioxidant that scavenges superoxide anion. Several studies suggest that mROS may be involved in OMA1 regulation or induce OMA1-dependent OPA1 proteolytic processing [43,44]. Based on these reports, we initially hypothesized that mROS induces OMA1-dependent OPA1 proteolytic processing during CS + RW. NRK cells stably overexpressing MnSOD, showed a threefold increase in MnSOD activity compared to control (Figure 4A). Interestingly, in both NRK and NRK cells overexpressing MnSOD, L-OPA1 was reduced after CS + RW (Figure 4B,C). OMA1 and S-OPA1 protein expression remained statistically unchanged across all conditions (Figure 4D,E). Taken together, these data show that overexpressing MnSOD in NRK cells does not prevent OMA1-dependent OPA1 proteolytic processing suggesting that mROS does not directly lead to increased OPA1 cleavage during CS + RW.

### 3.5. MnSOD Overexpression Attenuates CS + RW-Induced Mitochondrial Respiratory Dysfunction

**Figure 5:**
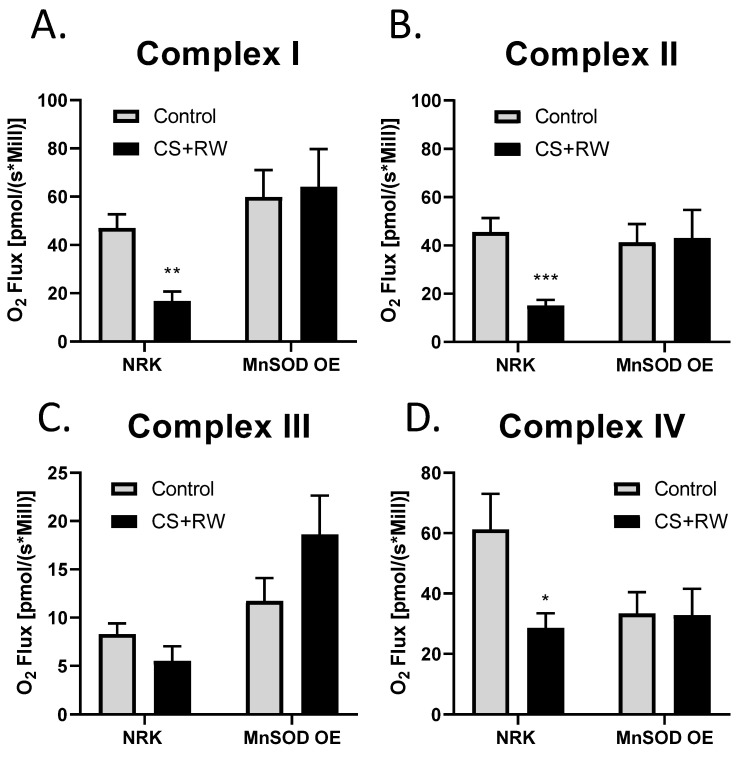
MnSOD overexpression mitigates CS + RW-induced mitochondrial respiratory dysfunction in NRK cells. High-resolution respirometry was used to measure mitochondrial respiration at electron chain complexes I–IV (**A**–**D**) in NRK cells and NRK cells overexpressing MnSOD. There was a statistically significant decrease in mitochondrial respiration in complex I, complex II and complex IV in CS + RW compare to control in NRK cells; however, this was prevented in NRK cells overexpressing MnSOD. Unpaired t-test was used to assess differences between conditions. Values are expressed as ±SEM (*n* = 7–11). * *p* < 0.05, ** *p* < 0.01 and *** *p* < 0.001.

High-resolution respirometry (HRR) was used to assess individual mitochondrial complex-dependent respiration in live NRK cells and NRK cells overexpressing MnSOD. We have previously reported that 18 h CS + 2 h RW impairs mitochondrial respiration in NRK cells [45]. To our knowledge, this is the first study to evaluate the impact that MnSOD overexpression in NRK cells on mitochondrial respiration at complexes I-IV using whole cells (not isolated mitochondria). After CS + RW, complex I, II, and IV showed a decrease 63%, 66%, and 53%, respectively, in oxygen flux compared to control NRK cells. Excitingly, NRK cells overexpressing MnSOD showed full protection against CS + RW-induced respiratory dysfunction at complexes I, II, and VI (Figure 5A–D). Taken together, these results suggest that overexpressing MnSOD restores mitochondrial respiratory function during CS + RW.

### 3.6. Overexpression of MnSOD Attenuates CS + RW-Induced Cell Injury

**Figure 6:**
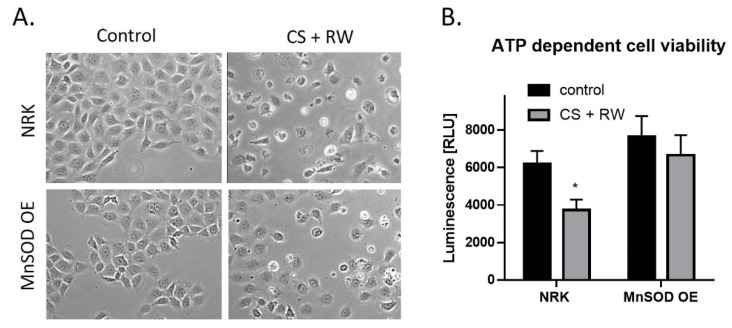
MnSOD overexpression attenuates CS + RW-induced cell death. (**A**) Microscopic evaluation for qualitative assessment of cell death. (**B**) ATP-dependent cell viability assay shows that overexpression of MnSOD protects against CS + RW induced loss of cell viability. Unpaired t-test was used to assess differences between conditions. Values are expressed as ±SEM (*n* = 5). * *p* < 0.05.

CS + RW-induced cell injury was evaluated qualitatively by microscopic evaluation and quantitatively by a commercially available kit, CellTiter-Glo luminescent cell viability assay. This assay measures cytotoxicity based on the quantification of metabolically active cells. Qualitatively, CS + RW resulted in abnormal cell morphology and cell death. However, in NRK cells overexpressing MnSOD, cell death was attenuated (Figure 6A). The luminescent cell viability assay revealed that CS + RW resulted in a 34% decrease in ATP-dependent cell viability compared to control NRK cells, which was blocked in MnSOD overexpressing cells (Figure 6B). Taken together, NRK cells overexpressing MnSOD mitigates CS + RW-induced cytotoxicity.

## 4. Discussion

There are three principle findings from the present study. We demonstrated for the first time that OMA1 plays a key role in proteolytically processing OPA1 (namely loss of L-OPA1) during CS + RW-induced renal cell injury. Secondly, mROS do not appear to be involved with the OMA1-dependent OPA1 proteolytic processing during CS + RW. Thirdly, mROS are involved with renal cell respiratory complex inhibition and cytotoxicity during CS + RW. Collectively, these studies suggest that enhanced OPA1 processing may not play a direct role in CS + RW induced mitochondrial and cell injury.

Both clinical and preclinical studies have shown that oxidative stress is implicated in renal injury during cold preservation/reperfusion [12,46,47,48,49]. In mammalian cells, MnSOD is the indispensable mitochondrial antioxidant enzyme that detoxifies the harmful free radical superoxide [50]. Oxidative stress has been involved in the pathophysiology of many diseases, including cancer, inflammatory diseases, diabetes, and neurodegenerative diseases [51]. ROS, which include superoxide and its reaction product, peroxynitrite, plays a significant role in tissue injury associated with ischemia and reperfusion during kidney transplantation. Our lab has extensively studied the role of MnSOD using renal tubular cells lines exposed to CS followed by rewarming (CS + RW) and employing rat models of renal CS followed by transplantation (CS + Tx) [17,19,33,45]. In earlier studies, we showed that transient overexpression of MnSOD confers protection from CS-induced renal injury [15] and MnSOD inactivation exacerbates renal ischemia/reperfusion-induced injury [52]. Similarly, adding MitoQ, a mitochondrial targeted antioxidant, to CS solution showed to be renoprotective using both in vitro and ex vivo renal models [16,17].

However, to our knowledge, this is the first study to evaluate the role of MnSOD with OPA1 proteolytic processing during CS. Unexpectedly, overexpression of MnSOD did not reverse OPA1-proteolytic processing. This finding was unexpected given there are many studies, suggesting that ROS leads to OMA1-dependent OPA1 proteolytic processing [27,53,54]. One possible explanation for the different outcomes between these earlier studies and the current study is the source of ROS. In most of these prior studies, cells were treated with acute (2–6 h) bolus additions of hydrogen peroxide as the ROS treatment, leading to enhanced OPA1 processing. In this situation, overexpression of MnSOD would be expected to have no effect since it could not scavenge the non-mitochondrial hydrogen peroxide. In our study, CS + RW was used to generate endogenous ROS over a 24 h time period, primarily originating from mitochondria based on our prior results [15,31,52]. Thus, it is possible that the lack of MnSOD–mediated protection against OPA1 processing observed in our study is because cytosolic ROS or stressors that do not originate from mitochondrial superoxide are needed to activate OMA1-mediated OPA1 processing. Future studies are clearly needed to pinpoint the mechanisms involved with OMA1-mediated OPA1 cleavage during CS + RW. Importantly, we observed that MnSOD overexpression in NRK cells protected against CS + RW-induced mitochondrial injury. NRK cells after CS + RW showed a significant decline in complex I, II, and IV. This finding is consistent with other studies, including Shrum et al., which also showed a decline in mitochondrial respiration [30]. Overexpression of MnSOD also mitigated CS + RW-induced cell death as indicated qualitatively by microscopic evaluation and restored ATP-dependent cell viability.

OPA1 exists in cells as a mixture of transmembrane L-OPA1 and soluble S-OPA1, which is generated by the proteolytic processing of OPA1. In this study CS + RW induces a significant loss of L-OPA1 and increase in S-OPA1 (Figure 2). Results from earlier studies suggested that L-OPA1 is competent for mitochondrial fusion and S-OPA1 is not [25,41,55]. However, more recent studies have shown that S-OPA1 is also capable of maintaining bioenergetics and cristae structure [55,56]. Interestingly, a new study by Lee et al. provides evidence that S-OPA1 generation improves cell survival under oxidative stress [57]. This new survival mechanism could play a role in our current findings, since we also see increased S-OPA1 during CS + RW, which is redox-independent suggesting that S-OPA1 may also have a protective role in the pathophysiology of renal CS + RW injury.

NRK cells exposed to 18 h CS alone or 18 h CS + 1, 3, 6 h RW led to a rapid loss of L-OPA1 and increased S-OPA1, suggesting a role for OMA1. We used two cell models designed to evaluate the role of OMA1 in this processing: NRK cells transfected with OMA1 siRNA during CS + RW and MEF OMA1 KO cells subjected to CS + RW. In both models when OMA1 was reduced, we observed blockade of the CS + RW induced loss of L-OPA1, suggesting that this is controlled in an OMA1-dependent manner. Interestingly, S-OPA1 was increased in NRK cells after CS + RW (Figure 2); however, there was no statistical change in S-OPA1 expression in NRK cells after transfection with OMA1 siRNA combined with CS + RW or in MEF WT and OMA1 KO subjected to CS + RW (Figure 3). These findings are consistent with other findings in models of renal injury where OMA1 mediates OPA1 proteolysis in experimental models of ischemic kidney injury and renal ischemia-reperfusion injury [40,58]. These data provide the possibility that transfection alone or loss of OMA1 may alter basal OPA1 proteolytic processing.

There are limitations to our renal cell CS in vitro model. However, the purpose of the current study was to expand upon our prior publication demonstrating extensive OPA1 cleavage following CS and transplantation using our clinically relevant rat kidney transplantation model [19]. Consistent with our in vivo data, exposing NRK cells to CS resulted in a very similar pattern of OPA1 cleavage, which partially validates the use of this cell model to investigate OPA1 regulation in more detail. The majority of published studies focused on OPA1 regulation use cell culture or yeast models whereby OPA1 levels are genetically modified and inducers of stress are non-physiological (bolus additions of hydrogen peroxide) to determine mechanisms. Uniquely, our study evaluated alterations to ‘endogenous’ OPA1 during renal cell CS, which provides a depiction on a physiological level compared to earlier studies.

## 5. Conclusions

**Figure 7:**
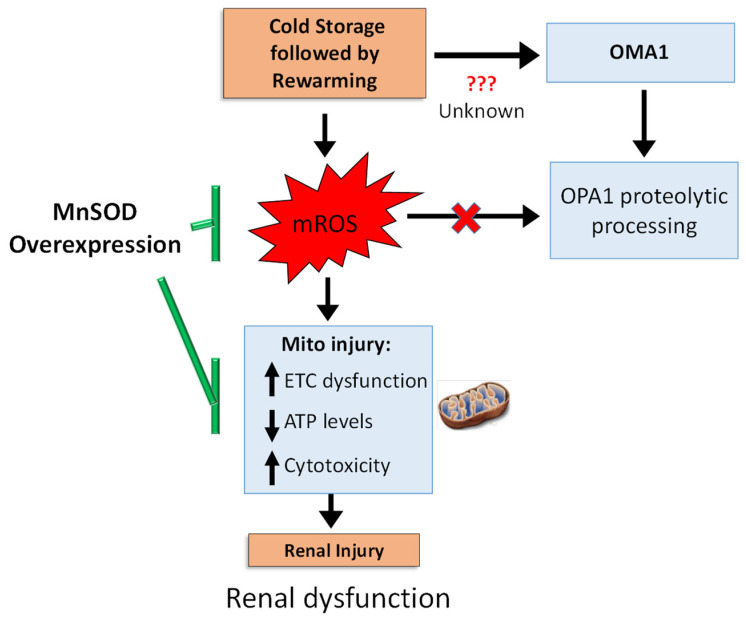
Summary schematic. CS + RW injury induces electron transport chain (ETC) dysfunction, decreased cellular ATP levels, and increases cytotoxicity, yet this injury is reversed by overexpressing MnSOD, suggesting a pivotal role of mROS. During CS + RW, OMA1 appears to be responsible for OPA1 proteolytic processing; but is independent of mROS. Overall, mROS is fundamental for reversing CS + RW-induced injury.

In summary, we propose the following schematic (Figure 7) CS + RW induces: (1) mROS, which subsequently leads to cytotoxicity by impairing mitochondrial respiration and decreasing ATP levels and (2) a mROS independent pathway leading to OMA1-depedent OPA1 proteolytic processing in NRK cells. Overexpressing MnSOD in NRK cells restored mitochondrial function and decreased cytotoxicity but did not reverse OPA1 processing, suggesting that OMA1-dependent OPA1 processing is mROS independent. Our study shows the involvement of more than one pathway, which encourages further dissection of pathways leading to altered OPA1 processing during CS + RW-induced renal injury. Since MnSOD overexpression prevented CS + RW-induced cytotoxicity, the utility of pharmacological targets designed to lower mROS should be further evaluated as novel therapeutics.

## Data Availability

Data is contained within the article.

## Abbreviations

CS	cold storage
mROS	mitochondrial reactive oxygen species
MnSOD	manganese superoxide dismutase
CS + RW	cold storage followed by rewarming
NRK cells	normal rat kidney proximal tubular cells
siRNA	small interfering RNA
HRR	high-resolution respirometry
ESKD	end-stage kidney disease
S-OPA1	short OPA1
L-OPA1	long OPA1
